# Recombinant human osteopontin expressed in *Nicotiana benthamiana* stimulates osteogenesis related genes in human periodontal ligament cells

**DOI:** 10.1038/s41598-017-17666-7

**Published:** 2017-12-11

**Authors:** Kaewta Rattanapisit, Supaniga Abdulheem, Daneeya Chaikeawkaew, Anchanee Kubera, Hugh S. Mason, Julian K-C Ma, Prasit Pavasant, Waranyoo Phoolcharoen

**Affiliations:** 1 Department of Pharmacognosy and Pharmaceutical Botany, Faculty of Pharmaceutical Sciences, Chulalongkorn University, Bangkok, Thailand; 2 Research Unit of Mineralized Tissue, Faculty of Dentistry, Chulalongkorn University, Bangkok, Thailand; 3 Department of Genetics, Faculty of Sciences, Kasetsart University, Bangkok, Thailand; 4 Biodesign Institute Center for Immunotherapy, Vaccines, and Virotherapy, and School of Life Sciences, Arizona State University, Tempe, AZ 85287-4501 USA; 5 The Institute for Infection and Immunity, St. George’s, University of London, London, UK

## Abstract

Tissue engineering aims to utilise biologic mediators to facilitate tissue regeneration. Several recombinant proteins have potential to mediate induction of bone production, however, the high production cost of mammalian cell expression impedes patient access to such treatments. The aim of this study is to produce recombinant human osteopontin (hOPN) in plants for inducing dental bone regeneration. The expression host was *Nicotiana benthamiana* using a geminiviral vector for transient expression. OPN expression was confirmed by Western blot and ELISA, and OPN was purified using Ni affinity chromatography. Structural analysis indicated that plant-produced hOPN had a structure similar to commercial HEK cell-produced hOPN. Biological function of the plant-produced hOPN was also examined. Human periodontal ligament stem cells were seeded on an OPN-coated surface. The results indicated that cells could grow normally on plant-produced hOPN as compared to commercial HEK cell-produced hOPN determined by MTT assay. Interestingly, increased expression of osteogenic differentiation-related genes, including *OSX*, *DMP1*, and *Wnt3a*, was observed by realtime PCR. These results show the potential of plant-produced OPN to induce osteogenic differentiation of stem cells from periodontal ligament *in vitro*, and suggest a therapeutic strategy for bone regeneration in the future.

## Introduction

Tissue engineering is a technology in medical therapeutics using bioactive substitutes for functional restoration of lost tissues and impaired organs^1^. Various proteins are necessary for tissue or organ engineering. These proteins function in regulating cellular behavior, the structure of many signaling molecules, and the fabrication of scaffolds^2^. However, there are several limitations to using proteins for this objective, such as source availability and stability, batch-to-batch variability, transmission of infecting organisms and immunogenicity^3,4^. Recombinant DNA technology used to expression foreign genes in convenient host systems can overcome these impediments.

Many recombinant proteins were reported to function in tissue engineering, such as bone matrix protein, collagens^5,6^, elastin^7,8^, and spider silk^9,10^. Presently, several sources for recombinant protein production are available, including *E. coli*, yeast, insect cells, mammalian cells, and plants. Among these platforms, the plant system is the most recently developed. One advantage of plants over other systems is eukaryotic post-translational modification, which may be necessary for the function of proteins used in tissue engineering processes^11^. Other advantages include lack of human pathogens, speed, low cost, and highly scalable manufacturing^11,12^.

OPN has a role in bone formation. Also known as secreted phophoprotein1 (SPP1), it is a highly phosphorylated glycoprotein that is a prominent component of the mineralized extracellular matrix of bone^13^. OPN contains an Arg-Gly-Asp sequence that is a major integrin-binding site and functions to support adhesion of bone cells to the mineralized matrix^14,15^. The molecular weight of OPN varies between 45–74 kDa, depending on the level of phosphorylation and glycosylation^16^. OPN is a soluble protein present in most body fluids. One study suggested that OPN plays a role in adhesion and movement of osteoblasts in bone^17^. Besides bone cells, OPN can be secreted by mesenchymal stem cells (MSC) and can be further up-regulated during the osteogenic differentiation of these cells^18^.

In this study, plants are used to produce human osteopontin (hOPN) as a model for recombinant proteins for tissue engineering. The recombinant hOPN was expressed transiently in *Nicotiana benthamiana* using geminiviral vectors. The hOPN was purified by Ni affinity chromatography and characterized for its structural conformation compared with commercial hOPN. The effect of plant-produced hOPN on cell cytotoxicity and osteogenic differentiation were also investigated.

## Results

### Transient expression of hOPN in *N. benthamiana* leaves

**Figure 1:**
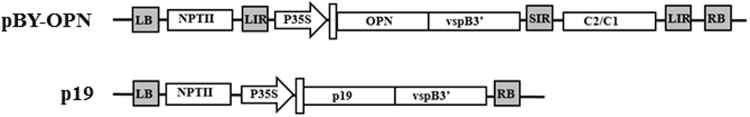
Schematic representation of the T-DNA regions of the vectors used in this study P35S: Cauliflower Mosaic Virus (CaMV) 35S promoter, *O. sativa* leader: *Oryza sativa* leader sequence, OPN: human osteopontin gene with 8X histidine residues at C-terminus, vspB3’: soybean vspB gene 3′ element, C2/C1: Bean Yellow Dwaft Virus (BeYDV) ORFs C1 and C2 which encode for replication initiation protein (Rep) and RepA, LIR: long intergenic region of BeYDV genome, SIR: short intergenic region of BeYDV genome, NPTII: expression cassette encoding nptII gene for kanamycin resistance P19: P19 gene from Tomato Bushy Stunt Virus (TBSV). LB and RB: the left and right borders of the Agrobacterium T-DNA region.

**Figure 2:**
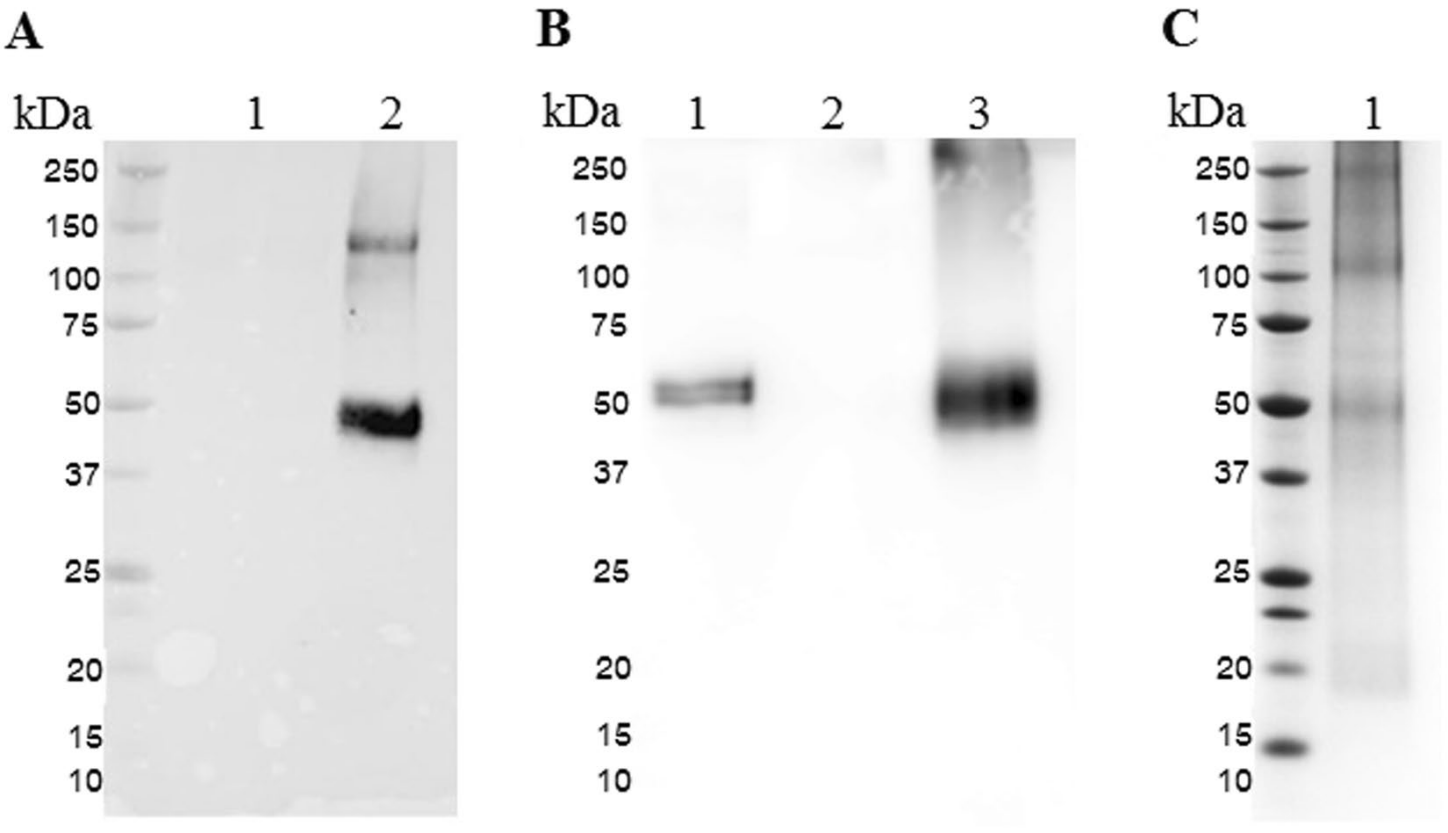
Western blot and SDS-PAGE of plant-produced hOPN. (**A**)Western blot of hOPN in crude extract of *N. benthamiana* leaf agroinfiltrated with pBY-OPN and pPS19 detected with mouse anti-human OPN and goat anti-mouse IgG conjugated with HRP. lane 1: wildtype *N. benthamiana*, lane 2: *N. benthamiana* agroinfiltrated with pBY-OPN and pPS19; (**B**) Western blot of purified hOPN from *N. benthamiana* detected with mouse anti-human OPN and goat anti-mouse IgG conjugated with HRP. lane 1: hOPN produced from HEK 293 cells, lane 2: wildtype *N. benthamiana*, lane 3: *N. benthamiana* agroinfiltrated with pBY-OPN and pPS19; (**C**) Purified plant-produced hOPN in SDS-PAGE. The numbers on the left are the size of the protein marker in kDa.

We produced hOPN by co-expression of hOPN (geminiviral vector pBY-OPN) with the gene silencing inhibitor p19^19^ from tomato bushy stunt virus, pPS19 (Fig. 1). The expression level of hOPN in *N. benthamiana* leaves was examined from 1 to 5 dpi by Western blot. The highest level of protein expression was found on day 3 (Supplementary Figure 1), up to ~100 ng hOPN per g leaf mass. Therefore, leaves were subsequently harvested on day 3 for protein extraction and purification. hOPN protein was observed at ~50 kDa by Western blot using anti-OPN antibody, while a negative control leaf extract showed no signal (Fig. 2A). The hOPN protein was purified from plant leaves using Ni affinity chromatography. The purified protein was confirmed by Western blot using an anti-OPN antibody (Fig. 2B) and SDS-PAGE (Fig. 2C).

### Secondary structure comparison by CD spectroscopy

**Figure 3:**
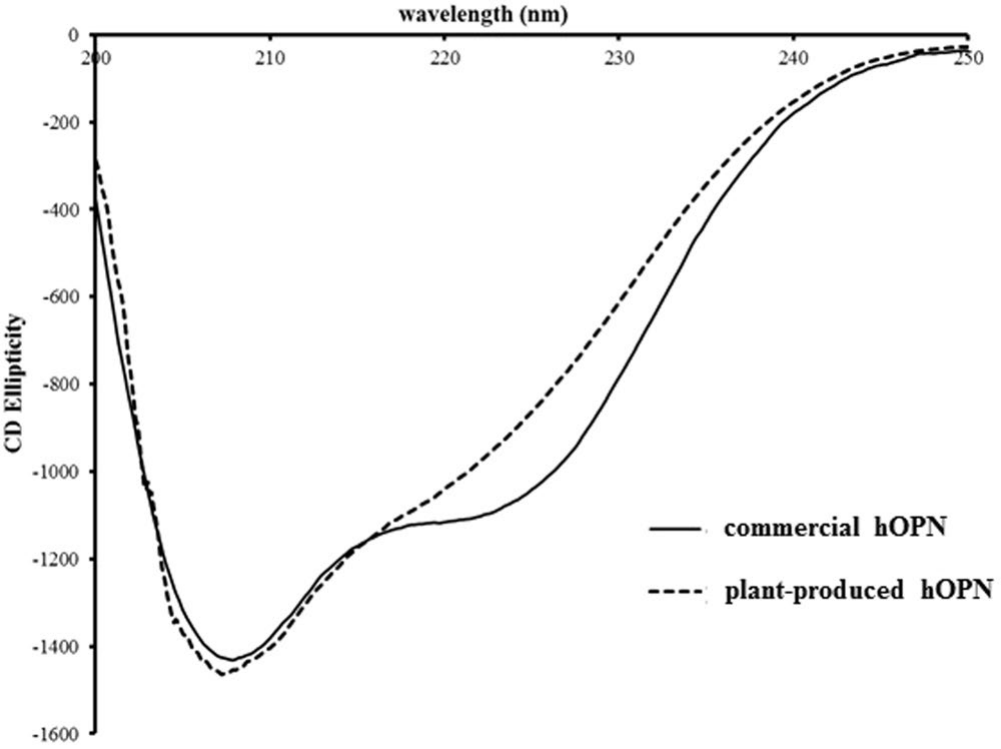
CD Structure analysis. Circular dichroism spectra of plant-produced hOPN and commercial hOPN (7.7 μM) in PBS (pH 7.4) were scanned by a circular dichroism spectrometer from 200–250 nm at. Both proteins exhibited maximum wavelength at 210 nm.

To verify the secondary structure, the CD spectra of commercial hOPN expressed in HEK 293 cells and plan-produced hOPN were monitored. The secondary structure contents of both proteins are showed in Table 1. The spectrum of both proteins had peaks at wavelengths of 208 nm and 222 nm (Fig. 3). These spectra suggested that both proteins contain similar secondary structures.

**Table 1:** The estimated secondary structure contents of commercial hOPN and plant hOPN.

Secondary structure contents (%)	hOPN produced from HEK 293 cells	hOPN produced from *N. benthamiana*
α-helix	2.5	8.3
β-sheet	34.3	28.1
turn	12.5	12.5
random	40.3	39.2
sum	89.7	88.1

### Tertiary structure comparison by fluorescence spectroscopy

**Figure 4:**
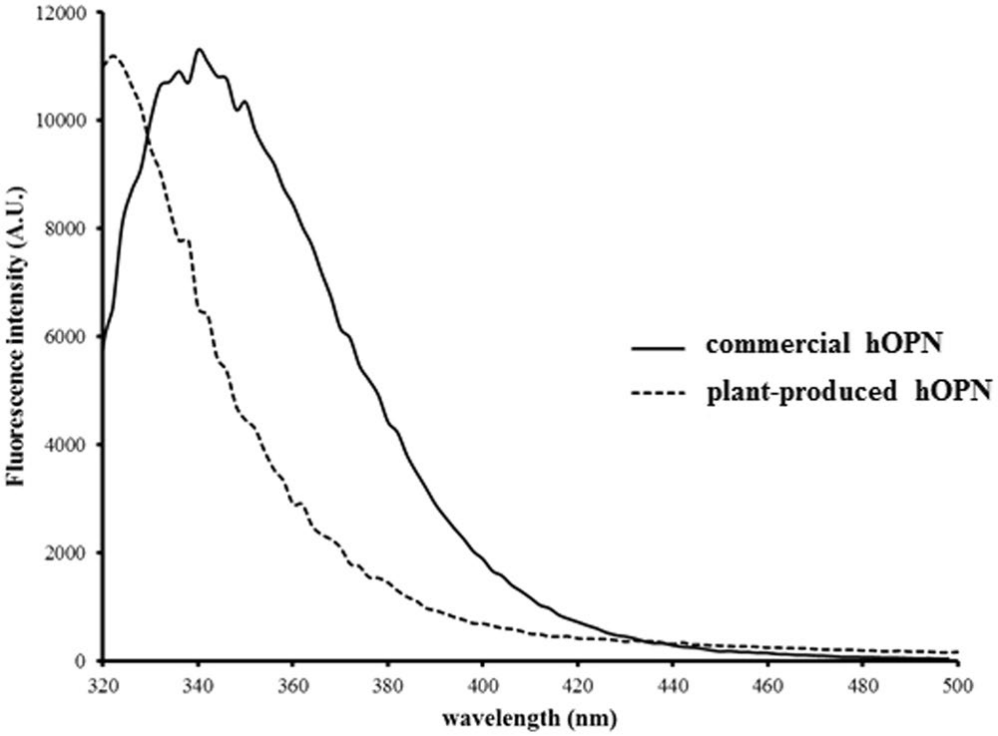
Intrinsic fluorescence spectra of commercial hOPN and plant-produced hOPN. Plant-produced hOPN showed blue shift of the maximum wavelength when compared to the commercial hOPN. The emission spectra were obtained from 320–500 nm, with the excitation wavelength at 280 nm.

The intrinsic tryptophan fluorescence spectra of commercial hOPN expressed in HEK 293 cells and plant-produced hOPN were characterized (Fig. 4). The maximum wavelengths of commercial hOPN and plant-produced hOPN were 340 and 320 nm, respectively. This result implied that the overall tertiary structure including the structure of glycosylation of these proteins were different.

### Effect of plant-produced hOPN on cell proliferation

**Figure 5:**
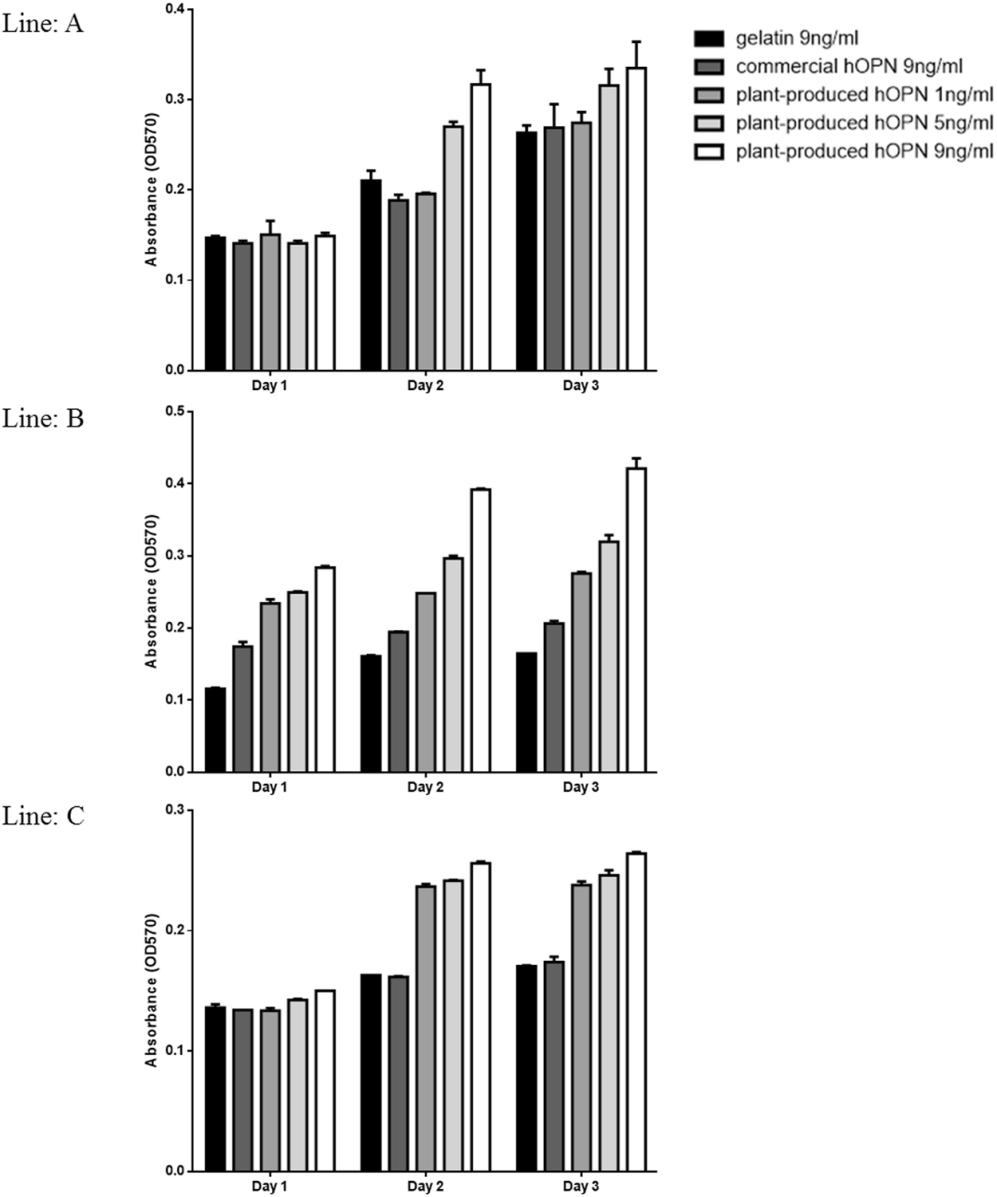
Effect of OPN on periodontal ligament cells (PDL) proliferation. Three PDL cell lines established from three different donors were cultured on gelatin-coated (9 ng/well), commercial hOPN-coated (9 ng/well) and plant-produced hOPN-coated surface (1. 5 and 9 ng/well) for 1–3 days. The experiments were done in triplicated. Cell survival was evaluated by MTT assay at 24 hours. Data represented the absorbance at 540 nm. Data are means of 3 independent replicate samples +/−SD. Control in this experiment is the untreated cells. (*p ≤ 0.05).

The effect of OPN on cell proliferation was examined by culturing hPDL cells established from 3 different donors on plant-produced hOPN-coated surface compared to cell culture on gelatin-coated and commercial hOPN-coated surfaces. The cell number was determined by MTT assay after cultured for 1, 2 and 3 days. The results showed that all 3 lines of hPDL cells could attach and grew normally on all surfaces tested (Fig. 5) indicating that none of the materials tested cytotoxic. However, cells cultured on 5 and 9 ng/ml of plant-produced hOPN grew significantly faster than when cultured on 9 ng/ml of gelatin on day 2 and day 3.

### Plant-produced hOPN activates osteogenesis related genes

**Figure 6:**
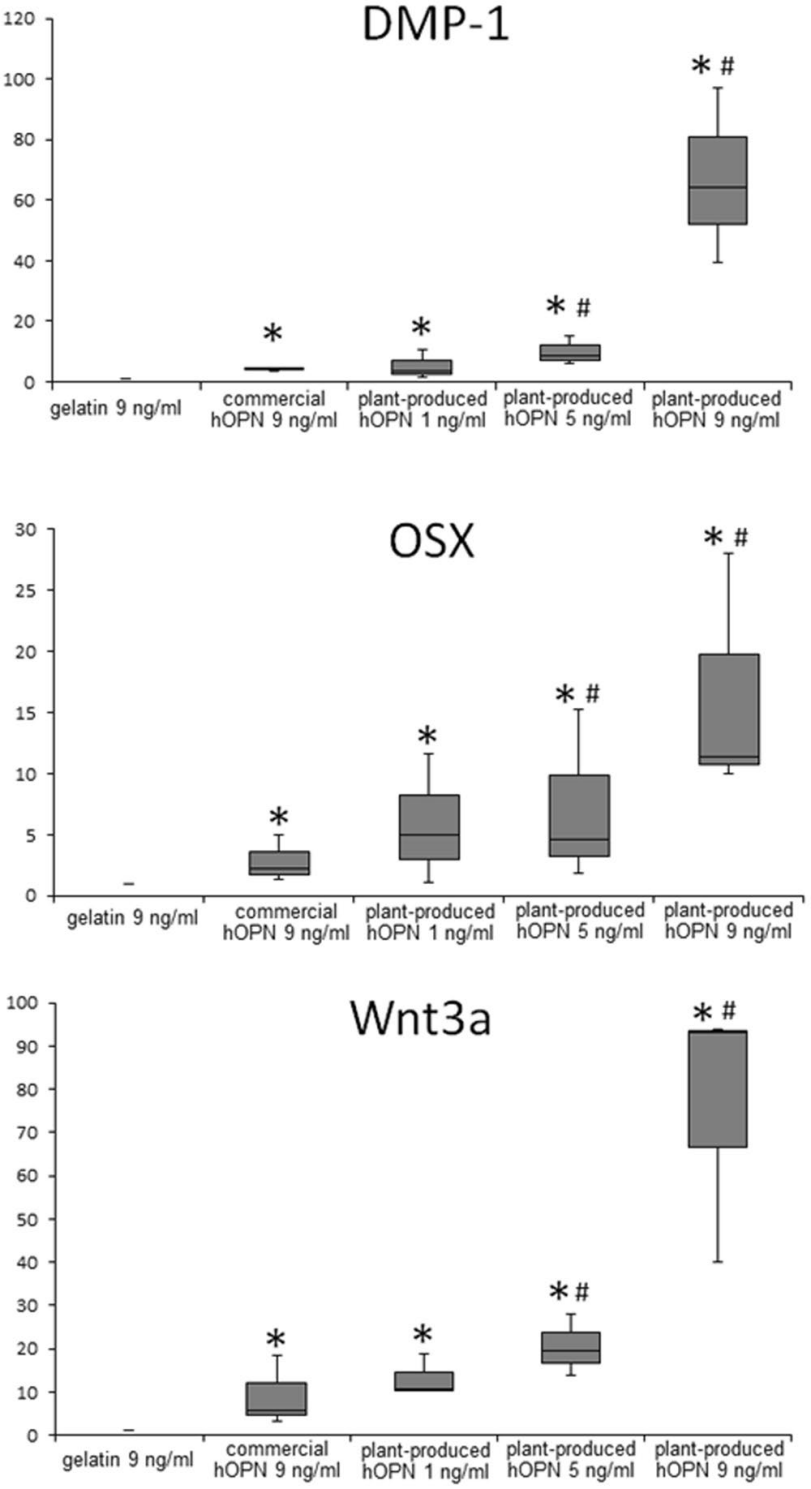
Plant-produced hOPN increased the mRNA expression of osteogenic markers. PDL cells from three different donors were treated with of 9 ng/ml gelatin, 9 ng/ml commercial hOPN, and 1, 5, 9 ng/ml plant-produced hOPN for 24 hours. Total RNA was extracted and real time PCR was performed using primer sets for human *OSX*, *DMP1*, and *Wnt3a* genes. Relative mRNA expression values were calculated normalized to the cells treated with gelatin. The values obtained for control gelatin were set at 1 for subsequent fold change calculation. (*p ≤ 0.05 compared to 9 ng/ml gelatin, ^#^p ≤ 0.05 compared to 9 ng/ml commercial hOPN).

Cells were treated with 1, 5 and 9 ng/ml of plant-produced hOPN or 9 ng/ml commercial hOPN for 72 hours. The mRNA was extracted and tested for the expression of *OSX*, *DMP1*, and *Wnt3a* genes by qRT-PCR. Figure 6 shows the expression level of these genes compared to cells cultured on gelatin with commercial OPN 9 ng/ml. The results from Fig. 6 indicated that commercial hOPN–coated surface could up-regulate the expression of *OSX*, *DMP1*, and *Wnt3a*. Interestingly, the mRNA expression of these three genes in cells seeded on plant-produced hOPN were significantly higher than those found in cells seeded on either gelatin-coated or 9 ng/ml commercial hOPN-coated surfaces.

## Discussion

In this study, we have demonstrated that functional matrix protein hOPN could be produced in *N. benthamiana*. The plant-produced hOPN was purified by Ni affinity chromatography and characterized by Western blot analysis and ELISA. The molecular weight of plant-produced hOPN was approximately 50 kDa, which is similar to that found in human tissue^20,21^. Our results also indicated that plant-produced hOPN contained similar epitopes as the protein synthesized by mammalian cells as judged by the ability to be recognized by a monoclonal anti-human OPN (Fig. 2B). Interestingly, plant produced hOPN showed increased biological function regarding osteogenic differentiation as compared to the same amount of recombinant hOPN from HEK cells (Fig. 6).

Recombinant hOPN was previously expressed in *E. coli* ^22–25^. Although hOPN produced from *E. coli* showed activity in cell adhesion, proliferation and differentiation of osteoblast cells^23^, it has been shown that the post-translational modifications of recombinant hOPN affects the biological activities related to the regulation of cell adhesion regulation^26^, which is necessary for bone regeneration. Therefore, a production platform that can provide effective post-translational modification is needed for recombinant hOPN production.

This is the first study showing that plants can be used as a platform to produce hOPN. The geminiviral replicon system has previously been used to produce different proteins such as antibody^27,28^, antigens^28^, and immune complex^29^. In this study, the geminiviral vector resulted in rapid transient expression - only 3 days after transfection. Although the expected mass of the unmodified hOPN protein is 33 kDa, posttranslational modifications increase the molecular weight to 45–75 kDa depending on conditions^30^. Our result showed that plant-produced hOPN is approximately 50 kDa (Fig. 2). OPN contains both N-linked and O-linked glycosylation sites^31^. Two N-glycan sites were found in OPN from bone, kidney tissues, macrophages, urinary stones and human milk^32^. Seven O-glycosylation regions were detected on hOPN, which are occupied by highly heterogeneous O-glycans^33^. A previous study demonstrated that the O-glycosylation of hOPN regulated its biological activities and affected the phosphorylation status^34^.

We used circular dichroism (CD) to evaluate the secondary structure of both commercial hOPN and plant-produced hOPN (Fig. 3). These data confirmed the similarity of secondary structures between hOPN produced from mammalian cells and plants. However, the intrinsic fluorescence spectra of these proteins were found to be somewhat different (Fig. 4). Since hOPN is a glycosylated protein, the different glycosylation patterns in human and plant may affect the overall protein conformation. This result is consistent with several reports that some proteins with different patterns of glycosylation showed shifted spectra^35,36^.

The biological activity of the purified plant-produced hOPN was further examined in human periodontal ligament stem cells. OPN has been shown to contain an RGD amino acid sequence that promotes cell attachment via integrin *alphavbeta3*. In this study, the ability to support cell growth and attachment was compared between gelatin, denatured type I collagen, commercial recombinant hOPN and plant-produced OPN using PDL cells. No significant differences were detected after 24 hours in culture. However, cells cultured on the plant produced hOPN-coated surface had significantly increased cell proliferation after 2 and 3 days in culture (Fig. 5). Interestingly, both types of hOPN, when immobilized on the cell surface using layer-by-layer technique, could significantly induce expression of osteogenic related genes such as *OSX, DMP1*, and *Wnt3a* (Fig. 6). Wnt3a is a the key protein to induce osteogenic differentiation of MSCs^37^, while osterix (OSX) is the key transcription factor that functions in osteogenic differentiation^38^. Dentine matrix acidic phosphoprotein-1 (DMP-1) is also one of the matrix proteins that has been recognized as a marker of mineralized tissue formation^39^. The ability of OPN to support osteogenic differentiation has been reported previously^39^. In that report, addition of a neutralizing antibody to OPN affected osteogenic differentiation of bone marrow mesenchymal stem cells. Our data showed the stimulation of these osteogenic related genes in cells isolated from three different donors. Three different donors are a minimum number for statistical analysis and were used in several reported studies^40–43^. Therefore, the plant-produced hOPN has clinical potential for supporting the osteogenic regeneration process.

It is also interesting to note that, at the same concentration, 9 ng/ml, the plant-produced OPN induced *DMP1, OSX*, and *Wnt3a* genes significantly better than commercial hOPN (Fig. 6) in all three established hPDL from 3 different donors, by a factor of 5–10 times. The levels of induction are different among the donor cells due to individual genetic variation. Although the exact mechanism is still unclear, it is possible that the plant-specific glycan pattern might be involved. Thus the glycan structures of plant-produced hOPN will be studied in detailed in future.

Our study firstly reported that the recombinant hOPN protein can be produced in plants by transient expression and the plant-produced hOPN can induce the genes involved in bone regeneration *in cellulo*. Further study of the plant-produced hOPN in an animal model is necessary to evaluate function in bone regeneration. Moreover, other proteins, which induce a different mechanism of bone regeneration, will also tested for expression in plants. The plant platform has potential to be a robust factory to produce low cost and effective recombinant proteins, which can facilitate the development of tissue engineering techniques that are affordable for patients in the future.

## Methods

### Expression vector construction

Human osteopontin gene^44^ was amplified using hOPN forward and hOPN reverse primers (Table 2). The vector containing human OPN was a gift from Professor Cecilia M. Giachelli. The PCR product was cloned into pGEM-T Easy (Promega, USA) for sequencing and cut with *Nco*I and *Sac*I restriction enzymes. The geminiviral vector pBY030.2R^45^ was cut with *Nco*I and *Sac*I and ligated with the digested hOPN gene to make pBY-OPN (Fig. 1). After verification, the plasmid was electroporated into *Agrobacterium tumefaciens* strain GV3101.

**Table 2:** Primers list.

Gene	Sequence (5′ to 3′)	Product size (bp)	Sequence ID
hOPN	Forward: CCATGGAACTTGGACTTTCTTGG	950	J04765.1
	Reverse: GAGCTCTTAATGATGGTGATGGTGGTGATGATG		
qGAPDH	Forward: CACTGCCAACGTGTCAGTGGTG	121	NM_002046.5
	Reverse: GTAGCCCAGGATGCCCTTGAG		
OSX	Forward: GCCAGAAGCTGTGAAACCTC	160	NM_152860.1
	Reverse: GCTGCAAGCTCTCCATAACC		
qDMP-1	Forward: ATGCCTATCACAACAAACC	213	NM_004407.3
	Reverse: CTCCTTTATGTGACAACTGC		
Wnt3a	Forward: CTGTTGGGCCACAGTATTCC	113	NM_033131.3
	Reverse: GGGCATGATCTCCACGTAGT		

### Plant inoculation and protein expression

*Nicotiana benthamiana* plants, 6 to 8 weeks-old, were co-infiltrated with two *Agrobacterium* cell lines that contained pBY-OPN or pPSp19 by vacuum infiltration^27^. Plants were maintained in cultured room with a 16 h light/8 h dark cycle at 28 °C after infiltration. The leaves were harvested on days 1, 2, 3, 4, and 5 post-infiltration (dpi) for expression time-course experiments. For other experiments, the leaves were harvested on day 3 dpi. Infiltrated tobacco leaves were homogenized by using a blender with extraction buffer (5 mM imidazole, 20 mM Tris-HCl pH 7.4, 50 mM NaCl). Crude extract was filtered through Miracloth and centrifuged at 26,000 *g* at 4 °C for 30 min. The supernatant was filtered with 0.2-micron filter and used for Ni affinity purification. Chelating Sepharose^TM^ (GE healthcare, UK) was packed into a column and washed with 10 bed volumes of distilled water. The resin was charged with 5 column volumes (CV) of 50 mM NiSO_4_.6H_2_O. After washing with 10 CV distilled water, the resin was washed with 10 CV of binding buffer (5 mM imidazole, 20 mM Tris-HCl pH7.4, 50 mM NaCl). The plant extract solution was loaded into the column. After washing with 10 CV washing buffer (20 mM imidazole, 20 mM Tris-HCl pH7.4, 50 mM NaCl), the purified protein was eluted with eluting buffer (250 mM imidazole, 20 mM Tris-HCl pH7.4, 50 mM NaCl) and analyzed by SDS-PAGE and Western blot.

### SDS-PAGE and Western blot

The proteins were denatured by boiling for 5 minutes with loading buffer (125 mM Tris-HCl, 12% (w/v) SDS, 10% (v/v) glycerol, 22% (v/v) β-mercaptoethanol, and 0.001% (w/v) bromophenol blue) and separated on 10% sodium dodecyl sulfate polyacrylamide gel electrophoresis (SDS–PAGE). Proteins were either visualized by Coomassie blue staining or electrophoretically transferred to polyvinlidene difluoride (PVDF) membrane (Amersham Hybond-ECL; Amersham Biosciences, UK). The membrane was blocked with 5% non-fat dried milk, 0.1% Tween20 in PBS (PBST). The membrane was probed with mouse monoclonal anti-OPN antiserum (Abcam, UK) diluted 1:5,000 in 1% non-fat dried milk in PBST and goat anti-mouse IgG-HRP conjugated (Sigma, USA) diluted 1:10,000 in 1% non-fat dried milk in PBST. The membranes were developed by chemiluminescence using ECL plus detection reagent (GE Healthcare, UK).

### Secondary structure characterization by circular dichroism spectroscopy

Circular dichroism (CD) spectra were recorded by Chirascan (Applied Photophysics, Ltd) to determine the secondary structure of recombinant hOPN expressed in HEK 293 cells (Sigma-Aldrich, USA) and plant-produced hOPN. The spectra were measured between 190 nm and 250 nm. The measurements were conducted using protein concentrations of 0.10 mg/mL in 10 mM potassium phosphate buffer (pH 7.4). All data presented are the means of three independent measurements. The secondary contents of both proteins were calculated using Raussens *et al*. method^46^.

### Intrinsic fluorescence spectroscopy

Emission spectra of commercial hOPN and plant-produced hOPN were monitored. The spectra were scanned from 300 to 500 nm using Spark 10 M multimode microplate reader (Tecan Group Ltd., Männedorf, Switzerland), based on an excitation of intrinsic fluorescence from aromatic side chains at 280 nm. Samples containing 0.10 mg/ml of protein were analyzed. Three repetitive scans were obtained and averaged.

### OPN quantification by ELISA

The purified plant-produced hOPN was quantified by ELISA. The protocol was done according to manuals of Human Osteopontin (OPN) ELISA Kit (Sigma-Aldrich, USA). The absorbance was measured using microplate reader at 450 nm.

### Cells

Human periodontal ligament (hPDL) cells were isolated and maintained according to a previous report^47^. The protocol was approved by the Ethics Committee, Faculty of Dentistry, Chulalongkorn University and all methods were performed in accordance with the relevant guidelines and regulations. The informed consents were obtained. Briefly, periodontal tissues were scraped from the middle one-third of the root surface the extracted third molars. The explants were cultured in Dulbecco’s modified Eagle’s medium containing 10% fetal bovine serum, 2 mM L-glutamine, 100 units/mL penicillin, 100 µg/mL streptomycin and 250 ng/mL amphotericin B at 37 °C in a humidified 5% carbon dioxide atmosphere until the cells were outgrown from the explants and routinely subcultured after reaching confluency. All cell culture reagents were purchased from Gibco BRL (Carlsbad, CA, USA). Cells were prepared from six donors, represented by line A, B, C, D, E, and F. All the experiments were done using cells from passage 3–5. To characterize the mesenchymal surface markers, flow cytometry was performed to determine the surface expression of CD45, CD73, CD90 and CD105 according to our previously published report^48,49^.

### Cell proliferation assay

MTT (3-(4,5-dimethylthiazol-2-yl)-2,5-diphenyltetrazolium bromide) (USB Corporation, USA) is a tetrazolium compound that will be reduced to a formazan product by mitochondrial dehydrogenase. The amount of formazan product represents the metabolic activity of viable cells at a particular time point. Thus, cell proliferation can be indirectly determined by the changes in the amount of formazan. Cells were seeded at density of 50,000 cells per wells in 24-well-plate and assay was performed at days 24, 48, or 72 hours. Cells were treated with 300 µl of 0.5 mg/ml MTT solution and the plate was incubated for 20 min at 37 °C. After that, the MTT solution was aspirated and the well washed with PBS. Then 500 µl of Glycine:DMSO (1:9) was added to each well. After the formazan crystals had dissolved, the absorbance was determined spectrophotometrically at 570 nm using a reference wavelength of 630 nm on an ELX800UV universal microplate reader (Bio-Tek Instruments Inc., Vermont, USA). The experiments were done in triplicate.

### Real-time PCR analysis for osteoblast differentiation markers

Total RNA was extracted from each experiment with Isol-RNA Lysis reagent (5Prime, Gaithersburg, MD, USA) and 1 µg of RNA per sample was converted to cDNA using a reverse transcriptase kit (Promega, Madison, WI, USA). Real-time quantitative polymerase chain reaction was performed using a Lightcycler Nano realtime polymerase chain reaction machine (Roche Applied Science, Indianapolis, IN, USA) using FastStart Essential DNA Green Master (Roche Applied Science). The PCR protocol was set as; denaturation at 94 °C for 10 seconds, annealing at 60 °C for 10 seconds, and extension at 72 °C for 10 seconds for 45 cycles. The reaction product of GAPDH was used as a reference gene for the internal control. The primer sequences are shown in Table 2.

### Statistical Analysis

All experiments were performed using cells isolated from three different donors. Statistical evaluation was performed using SPSS 16.0 software (SPSS, USA). The Mann Whitney U test was employed for two group comparison and the Kruskal Wallis test followed by pairwise comparison was used for comparing three or more groups. A significant difference was considered at p ≤ 0.05.

## Electronic supplementary material

Supplementary information
