# Supplementary material for: Recombinant human osteopontin expressed in Nicotiana benthamiana stimulates osteogenesis related genes in human periodontal ligament cells
Source: Sci Rep. 2017 Dec 11;7:17358. doi: 10.1038/s41598-017-17666-7 (PMC5725595; doi:10.1038/s41598-017-17666-7)

**Recombinant human osteopontin expressed in *Nicotiana benthamiana* stimulates osteogenesis related genes in human periodontal ligament cells**

Kaewta Rattanapisit^1^, Supaniga Abdulheem^2^, Daneeya Chaikeawkaew^2^, Anchanee Kubera^3^, Hugh Mason^4^, Julian Ma^5^, Prasit Pavasant^2^, Waranyoo Phoolcharoen^1*^

^1^ Department of Pharmacognosy and Pharmaceutical Botany, Faculty of Pharmaceutical Sciences, Chulalongkorn University, Bangkok, Thailand

^2^ Research Unit of Mineralized Tissue, Faculty of Dentistry, Chulalongkorn University, Bangkok, Thailand

^3^ Department of Genetics, Faculty of Sciences, Kasetsart University, Bangkok, Thailand

^4^ Biodesign Institute Center for Immunotherapy, Vaccines, and Virotherapy, and School of Life Sciences, Arizona State University, Tempe, AZ, 85287-4501, USA

^5^ The Institute for Infection and Immunity, St. George’s, University of London, London, UK

**Supplementary Figure 1** Western blot of hOPN in crude extract of *N. benthamiana* leaf agroinfiltrated with pBY-OPN and pPS19 on day 1 to 5 dpi detected with mouse anti-human OPN and goat anti-mouse IgG conjugated with HRP.


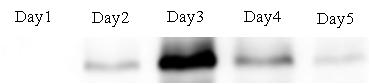

Supplement: Supplementary file 1 — Supplementary information [file 41598_2017_17666_MOESM1_ESM.doc]
